# 100 Million Views of Electronic Cigarette YouTube Videos and Counting: Quantification, Content Evaluation, and Engagement Levels of Videos

**DOI:** 10.2196/jmir.4265

**Published:** 2016-03-18

**Authors:** Jidong Huang, Rachel Kornfield, Sherry L Emery

**Affiliations:** ^1^ Health Media Collaboratory Institute for Health Research and Policy University of Illinois at Chicago Chicago, IL United States; ^2^ School of Journalism and Mass Communication University of Wisconsin-Madison Madison, WI United States

**Keywords:** electronic cigarettes, electronic nicotine delivery systems, ENDS, tobacco products, YouTube, social media

## Abstract

**Background:**

The video-sharing website, YouTube, has become an important avenue for product marketing, including tobacco products. It may also serve as an important medium for promoting electronic cigarettes, which have rapidly increased in popularity and are heavily marketed online. While a few studies have examined a limited subset of tobacco-related videos on YouTube, none has explored e-cigarette videos’ overall presence on the platform.

**Objective:**

To quantify e-cigarette-related videos on YouTube, assess their content, and characterize levels of engagement with those videos. Understanding promotion and discussion of e-cigarettes on YouTube may help clarify the platform’s impact on consumer attitudes and behaviors and inform regulations.

**Methods:**

Using an automated crawling procedure and keyword rules, e-cigarette-related videos posted on YouTube and their associated metadata were collected between July 1, 2012, and June 30, 2013. Metadata were analyzed to describe posting and viewing time trends, number of views, comments, and ratings. Metadata were content coded for mentions of health, safety, smoking cessation, promotional offers, Web addresses, product types, top-selling brands, or names of celebrity endorsers.

**Results:**

As of June 30, 2013, approximately 28,000 videos related to e-cigarettes were captured. Videos were posted by approximately 10,000 unique YouTube accounts, viewed more than 100 million times, rated over 380,000 times, and commented on more than 280,000 times. More than 2200 new videos were being uploaded every month by June 2013. The top 1% of most-viewed videos accounted for 44% of total views. Text fields for the majority of videos mentioned websites (70.11%); many referenced health (13.63%), safety (10.12%), smoking cessation (9.22%), or top e-cigarette brands (33.39%). The number of e-cigarette-related YouTube videos was projected to exceed 65,000 by the end of 2014, with approximately 190 million views.

**Conclusions:**

YouTube is a major information-sharing platform for electronic cigarettes. YouTube appears to be used unevenly for promotional purposes by e-cigarette brands, and our analyses indicated a high level of user engagement with a small subset of content. There is evidence that YouTube videos promote e-cigarettes as cigarette smoking cessation tools. Presence and reach of e-cigarette videos on YouTube warrants attention from public health professionals and policymakers.

## Introduction

YouTube is the third most visited site on the Internet [1] with a large and growing youth presence [2]. It provides a platform for individuals to upload, view, share, and respond to videos. While YouTube was intended for original, user-generated content, it also has become an important avenue for individuals and companies to reach wide audiences and market products, including tobacco products [3,4]. Prior studies have analyzed strategies of tobacco promotion on YouTube [5-7] as well as public attitudes toward tobacco products, finding that pro tobacco messages are prevalent and easy accessible relative to antismoking messages [8-10]. Videos related to electronic cigarettes and other alternative tobacco products (eg, little cigars, cigarillos, waterpipes, and smokeless tobacco) may portray these products in a particularly positive light [11-14]. Informational and promotional content available to consumers via social networks such as YouTube has the potential to shape how tobacco products are perceived and used, and thus has major public health implications [9,15,16].

YouTube may also be an important medium for spreading information and promotional messages about electronic nicotine delivery systems (ENDS) or *e-cigarettes*, which have increased rapidly in popularity and are heavily marketed and promoted online [17-19]. These products have raised controversy among the public health community; some evidence indicates their harm-reduction potential while others argue they may sustain nicotine dependence without reliably supporting tobacco cessation [20]. A 2010 US district court decision blocked e-cigarette manufacturers from marketing e-cigarettes as quit-smoking devices [21]. However, advertisers use indirect tactics such as affiliate marketing to circumvent that decision [10,22]; claims about the products’ health and safety profile and their role in smoking cessation may be commonplace on social networks despite the ruling [18,23]. E-cigarette companies and retailers may use YouTube to engage with customers and to disseminate largely unmonitored promotional messages. E-cigarette users (or *vapers*) may use YouTube to share experiences and advice regarding e-cigarettes, to organize around e-cigarette advocacy, and to engage in affiliate marketing [24]. YouTube is particularly suited for e-cigarette promotion and marketing because it allows subscribers to easily post content, including text, audio, and visual content, across several channels. Users can also easily interact with one another by subscribing to each other’s YouTube channels [25].

Little is known about the extent and type of e-cigarette content on YouTube and the level of engagement with such content. One 2009 study of smoking imagery on YouTube found that, of the most popular videos retrieved for cigarette-related keywords, e-cigarette videos represented approximately 8%, and these tended to include branding or claims about health benefits of e-cigarettes in comparison to cigarettes [9]. However, the number of e-cigarette videos included in this study was small (n=9) and e-cigarette popularity has since increased rapidly [17]. Another study compared typical usage patterns (eg, puff duration) between electronic and traditional cigarettes represented in YouTube videos, finding that e-cigarette users take longer puffs than conventional cigarette users, perhaps to compensate for low or unreliable levels of nicotine [26]. A recent study found that, among all UK Top 40 YouTube music videos over a 12-week period in 2013/2014, electronic cigarette branding appeared in 1% (95% CI 0-3) of videos [4]. Another recent study examined the top 20 YouTube search results using a number of e-cigarette-related keywords—196 videos in total—and found these videos to be overwhelmingly *pro* e-cigarettes (94%). In addition, this study found that the top three most prevalent genres among these videos were advertising, user sharing, and product reviews. A total of 84.3% of *pro* videos contained Web links for e-cigarette purchase. A total of 71.4% of *pro* videos claimed that e-cigarettes were healthier than conventional cigarettes. However, this study did not characterize the overall extent or type of e-cigarette content on YouTube [13]. Studies of tobacco-related videos on YouTube have typically relied on samples of top search results rather than quantifying the total number of relevant videos [4,5,7,9,11-14,27,28].

To date, tobacco control policies explicitly regulating e-cigarettes have been enacted only at the state [29] and municipal [30] levels. In April 2014, the US Food and Drug Administration (FDA) proposed a deeming rule that would extend its regulatory authority over tobacco products to include electronic cigarettes. The proposed rule also bans the sale of e-cigarettes to minors and requires those purchasing the products to show proof of age [31]. The FDA has not yet addressed regulations on e-cigarette marketing on television, radio, and/or social media. However, should the deeming rule become finalized, the agency may propose additional rules restricting e-cigarette marketing and promotion. Understanding how YouTube—one of the top social media platforms—is used to promote and discuss e-cigarettes can clarify the potential impact of such discussion on consumers’ vaping-related attitudes and behaviors, a priority research topic identified by the recent National Institutes of Health (NIH) Electronic Cigarette Workshop [32], and also may suggest appropriate ways to regulate social media marketing for e-cigarettes and other tobacco products.

This paper characterized the overall extent and type of e-cigarette relevant content and level of engagement with that content on YouTube as of June 30, 2013, and predicted the number of e-cigarette videos that existed as of December 31, 2014. The research employed an automated YouTube crawling program, ContextMiner [33], to approximate the total number of videos related to e-cigarettes by continually compiling daily search results for a list of e-cigarette-related keywords. We used metadata for relevant videos to describe trends in video posting and engagement, and to assess the fraction of videos for which uploader-provided text fields—titles, descriptions, and tags—include discussion of smoking cessation, health, or safety; link to websites; or mention specific brands or component parts.

## Methods

### Retrieving E-Cigarette-Related YouTube Videos

This study employed a YouTube crawling program, ContextMiner [33], to retrieve the videos related to e-cigarettes available on YouTube as of June 30, 2013. To identify e-cigarette-related videos, we first compiled a list of 70 e-cigarette keyword rules (see Multimedia Appendix 1). Our keyword rules were developed through expert consensus and were expanded via an iterative process wherein an initial set of limited keywords, such as *e cig* and *electronic cigarettes*, were used to retrieve content and identify co-occurring words, which were then tested for relevance and added to form new keyword rules [34]. For each keyword rule, daily YouTube crawls were performed for 1 year between July 1, 2012, and June 30, 2013; metadata—title, description, tags, channels, posting date—for matching videos were retrieved and downloaded to a database. Videos were retrieved on the basis of YouTube's *relevance* algorithm, which ranks videos in descending order of presumed relevance for a given keyword query. Since YouTube limited the number of videos retrieved by each crawl, we also conducted separate crawls where matching videos were retrieved on the basis of posting dates. Through the combination of two YouTube search techniques—relevance and posting date—and repeated daily crawls to overcome YouTube limits, we built a set of videos that YouTube’s algorithm deemed related to our e-cigarette keywords.

### Deduplicating and Refining

While the YouTube Web interface provides an approximation of the number of videos retrieved for a keyword search (eg, typing *electronic cigarettes* into the YouTube search bar yielded 89,800 results on April 8, 2015), this number is an approximation and may include numerous irrelevant videos. We reviewed our YouTube e-cigarette video database and excluded duplicate videos using the YouTube video ID, a unique identifier assigned by YouTube. Deduplication yielded 42,484 unique videos retrieved by one or more of our keyword rules over the study period. Unique videos were then reviewed to assess relevance of each video to electronic cigarettes. During this process, we noted that our database included many videos—mainly music videos—that did not contain e-cigarette-related keywords in any collected metadata fields and that did not include e-cigarette content in the videos themselves. This finding likely reflects that YouTube’s search algorithms incorporate additional metadata not collected in our crawls, and possibly indicates that some videos were uploaded in order to boost the relevance of related videos (ie, posted in response to e-cigarette videos) [35]. To exclude videos not relevant to e-cigarettes, we used a two-step method. First, two coders viewed the top 50 most viewed videos in our database to assess whether they were relevant to e-cigarettes. The reason for manual review of the top 50 most viewed videos was to ensure that our view calculation was not artificially inflated by nonrelevant videos, which tended to be associated with higher view counts. Five videos were deemed irrelevant by both coders during this process. In the second step, we searched metadata fields—description, tags, and title—using a list of e-cigarette-specific terms (see Multimedia Appendix 2) and classified relevant videos as those that contained such terms in their metadata fields. Our keyword algorithm was highly accurate in discerning between relevant and irrelevant content in a random sample of 500 retrieved videos, with 95% of allocations agreeing with a human coder. Approximately one-third (34%) of videos were classified as irrelevant in this step and were excluded from our database. The final database contained 28,089 e-cigarette-relevant videos.

### Types of Metadata

For all e-cigarette-related videos in our database, two main types of metadata were collected: static video characteristics and, where available, dynamic engagement data. Static video characteristics included video title and description, any tags the uploader provided, YouTube channel with which the video was associated (eg, *Tech*, *HowTo*, and *Entertainment*), posting date, name of uploader account, and video URL. Dynamic daily engagement data included view count, number of comments, number of ratings, and average rating.

### Overall Presence and Content Coding

#### Measuring Engagement With Videos

Metadata associated with each video were used to describe time trends in video posting and viewing on YouTube and to tabulate the total number of views, comments, and ratings as of June 30, 2013. Simple linear extrapolations were used to project time trends in video posting and viewing predicted to occur from July 2013 to December 2014.

#### Content Coding

Video titles, descriptions, and tag fields were searched to assess the frequency with which they mentioned several themes of interest to informing policy and public health: health-related themes, safety themes, smoking cessation themes, promotional offers, Web addresses, product types (eg, e-hookah and e-liquid), or specific top-selling brands [36]. We also searched metadata fields for names of celebrities known to have promoted or demonstrated electronic cigarette use [37,38], such as actress Katherine Heigl who smoked one on The Late Show with David Letterman.

We conducted searches via the YouTube search interface among our collected videos to identify accounts that appeared affiliated with the top 10 e-cigarette brands. These were identified by retail store sales via the Nielsen store scanner data [36] on the basis of brand-related account names and links to official brand websites on account pages. For these accounts, we tabulated the number of videos posted, number of subscribers, and the total view count.

#### Age Restrictions

We examined the existence of age restrictions for e-cigarette YouTube videos on a simple random sample of 280 videos (1%) from our database. Those videos were viewed from a Web browser cleared of previous browsing history and other identifiers, such as cookies and plug-ins, to determine any age restrictions applied by YouTube (ie, whether log-in was required to access content).

## Results

**Figure 1:**
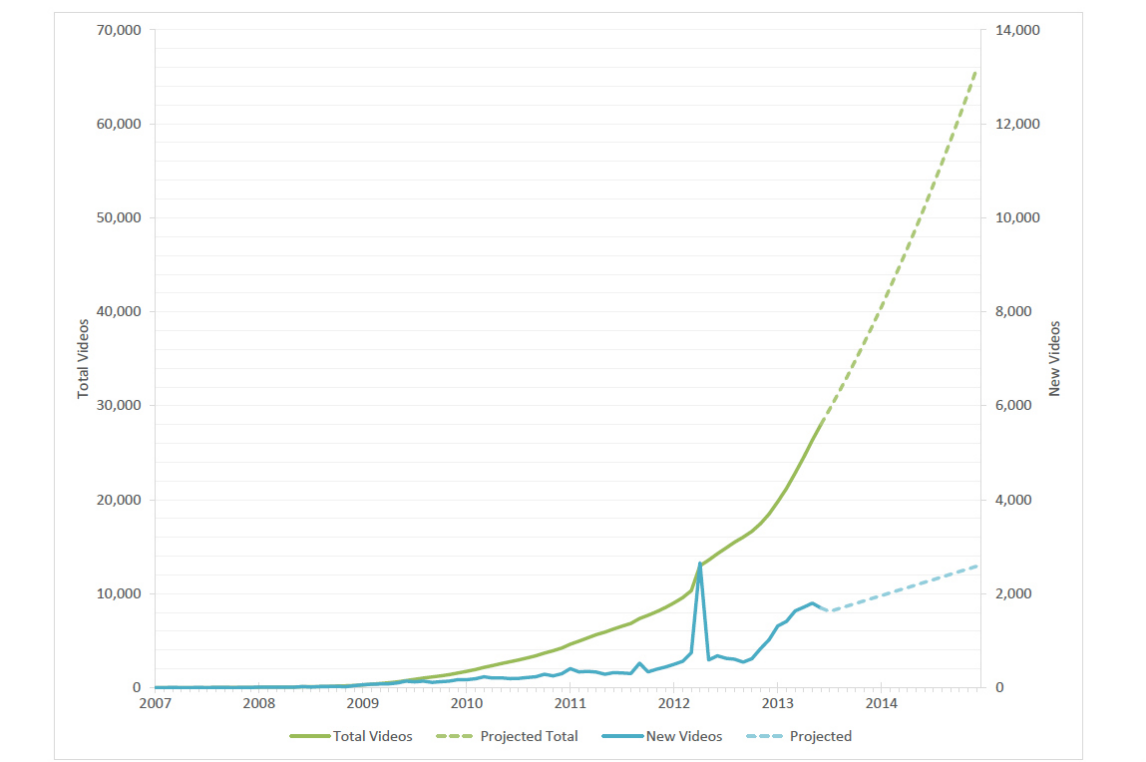
Monthly uploads of electronic cigarette-related videos from January 2007 to December 2014.

The first electronic cigarette videos in our sample were posted to YouTube in early 2007. The rate of posting increased over the study period from several new videos per month in 2007 to over 100 per month by late 2009, over 1000 per month by late 2012, and close to 2000 per month by June 2013 (see Figure 1). By the end of June 2013, there were 28,089 unique e-cigarette videos available on YouTube. We projected that the number of e-cigarette videos on YouTube would exceed 65,000 by December 2014, with more than 2500 new e-cigarette videos posted on the platform every month. No seasonal trends in posting rate were observed, although we observed a spike in posting in April 2012 when more than 2500 e-cigarette-related videos were posted—more than three times the monthly average for 2012. Over 80% of these videos mentioned the website EcigsFreeTrialOffer.com in video description fields. However, as of publication of this paper, the website is no longer active.

**Figure 2:**
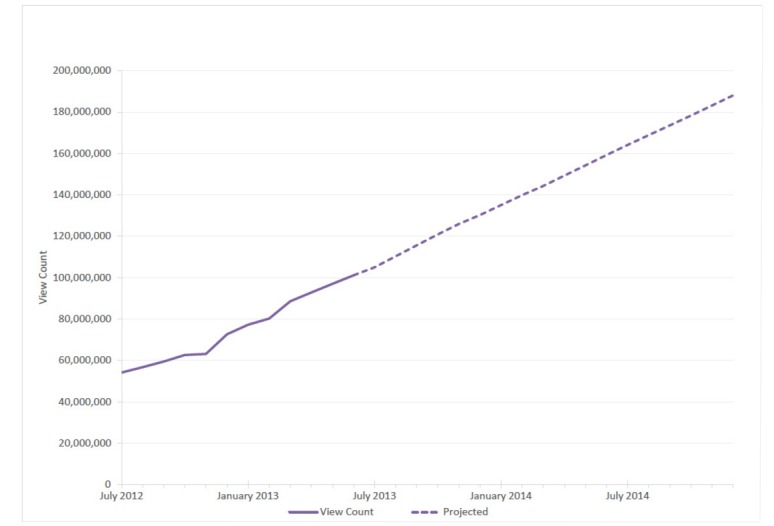
Total view counts for electronic cigarette-related videos from July 2012 to December 2014. View counts DO NOT include approximately 16% of videos with incomplete engagement data.

For approximately 85% of e-cigarette videos where dynamic engagement data were available, we plotted the trend in total views (see Figure 2). Combined view counts for e-cigarette videos in our database nearly doubled over the study period, increasing at a rate of approximately 4 million views per month, from 54 million in July 2012 to over 101 million by June 2013. By December 2014, total view counts of e-cigarette videos on YouTube were projected to exceed 188 million.

The 28,089 electronic cigarette videos identified in this study were posted by 9756 unique YouTube accounts (see Table 1). Posting was concentrated among a number of highly active accounts, with the top 1% of users posting 22% of videos in the sample. View count was even more highly concentrated, with 1% of videos accounting for 44% of total views. The most viewed video, with over 2.3 million views, was the music video *Life is a Roller Coaster* by Ronan Keating; there was no mention of e-cigarettes in the video itself, but the description field advertised an electronic cigarette retailer and the video tags mentioned several top e-cigarette brands. The second most viewed video, with 1.7 million views, was a UK television advertisement for E-lites, an e-cigarette brand. In total, the included videos had almost 101 million views and garnered over 380,000 ratings and over 280,000 comments.

Nearly all of the videos were classified under five YouTube categories (see Table 2): *People* (7610/28,089, 27.09%), *Tech* (6279/28,089, 22.35%), *HowTo* (5470/28,089, 19.47%), *Entertainment* (2891/28,089, 10.29%), and *Education* (2879/28,089, 10.25%).

Table 3 summarizes our content coding. A total of 13.63% (3828/28,089) of video tags, titles, or descriptions referenced health; 10.12% (2842/28,089) referenced safety; and 9.22% (2591/28,089) referenced smoking cessation. A total of 11.06% (3108/28,089) mentioned discounts. The majority of videos included Web addresses (19,694/28,089, 70.11%). The most common website we identified was EcigsFreeTrialOffer.com (2257/28,089, 8.04%) (no longer active), followed by youtube.com (718/28,089, 2.56%), v2cigs.com (388/28,089, 1.38%), facebook.com (334/28,089, 1.19%), and E-Cig-Reviews.com (307/28,089, 1.09%). Metadata for 42.57% (11,957/28,089) of videos mentioned reviews, although these videos had a lower-than-average view count. Mentions of e-cigarette component parts were also common: 27.19% (7637/28,089) mentioned e-liquid, 14.05% (3946/28,089) referenced *mods* (ie, modifications), 12.93% (3631/28,089) referenced atomizers, and 11.88% (3338/28,089) referenced batteries. Blu was the most mentioned brand, occurring in metadata for 3507/28,089 videos (12.49%). NJOY mentions were present for 1235 out of 28,089 videos (4.40%), and the remaining top 10 best-selling brands were associated with fewer than 300 videos each. We tabulated frequency of mentions for additional brands that had high occurrence in the dataset and for two brands recently introduced by tobacco companies—Vuse by RJ Reynolds and MarkTen by Altria. We found frequent mentions of eGo (3103/28,089, 11.05%), V2 (2783/28,089, 9.91%), and Joyetech (2395/28,089, 8.53%). Vuse and MarkTen were associated with very low frequency.

Table 4 summarizes official YouTube accounts associated with top-selling [36]—based on retail store scanner data—e-cigarette brands in the United States. While the number of videos posted by official e-cigarette company accounts is small, the view counts are quite high. For example, although Blu’s YouTube account had posted only 32 videos as of June 2013, those videos have garnered close to half a million views.

In a sample of 280 random videos, none was age restricted by YouTube, although 2 (0.7%) did include age-related disclaimers at the beginning of video content. All but 2 videos (278/280, 99.3%) included mentions or images of e-cigarettes in video content; the remaining 2 videos (0.7%) mentioned e-cigarettes in text fields only.

**Table 1:** Characteristics of e-cigarette-related YouTube videos as of June 30, 2013.

Video characteristics	Value
Total videos, n	28,089
Posting accounts, n	9756
Average videos/account, mean (SD)	2.88 (8.81)
Average video duration (minutes), mean (SD)	4.97 (8.34)
Total view count, n	106,963,322
Average view count, mean (SD)	3967 (29,350)
Total number of ratings, n	380,075
Average ratings/video, mean (SD)	14.1 (99.7) (range 0-7024)
Average rating (1-5)	4.5
Total comments, n	282,020
Average number of comments, mean (SD)	10.5 (58.1) (range 0-3969)

**Table 2:** Categories of e-cigarette-related YouTube videos with metadata as of June 30, 2013.

Category^a^	Videos (n=18,103), n (%)
People	7610 (27.09)
Tech	6279 (22.35)
HowTo	5470 (19.47)
Entertainment	2891 (10.29)
Education	2879 (10.25)
Film	825 (2.94)
News	804 (2.86)
Comedy	587 (2.09)
Nonprofit	200 (0.71)
Music	190 (0.68)
Autos	81 (0.29)
Sports	77 (0.27)
Travel	73 (0.26)
Animals	66 (0.23)
Games	51 (0.18)
Shows	6 (0.02)

^a^Categories are mutually exclusive.

**Table 3:** Content of metadata for e-cigarette-related videos on YouTube as of June 30, 2013.

Content category		Search query	Videos (N=28,089), n (%)	View count (N=106,963,322), n (%)
**Claims and promotions**				
	Health	"*health*"	3828 (13.63)	15,298,094 (14.30)
	Safety	"*safe*"	2842 (10.12)	7,915,121 (7.40)
	Cessation	"*quit sm*", "*stop sm*", "*cold turkey*", "*give up sm*", "*quitting sm*", "*quitsmok*", "*cessation*"	2591 (9.22)	9,956,254 (9.31)
**Any health, safety, or cessation**			7036 (25.05)	25,547,563 (23.88)
	Ban	"* ban *", "*banned*", "ban *", "* ban,*", "ban,*"	484 (1.72)	3,925,388 (3.67)
	Discount	"*discount*", "*coupon*"	3108 (11.06)	8,297,899 (7.76)
	Free trial	"*free trial*", "*freetrial*"	2641 (9.40)	1,236,881 (1.16)
	Web address	"*http*", "*.com*"	19,694 (70.11)	74,142,105 (69.32)
				
**Type of video**				
	Review	"*review*"	11,957 (42.57)	36,075,723 (33.73)
	Demo	"*demo*", "*how to*", "*howto*"	2673 (9.52)	15,255,278 (14.26)
	Celebrity	Like "*katherine heigl*", Or Like"*stephen dorff*", Or Like "*bruno mars*", Or Like "*courtney love*"	153 (0.54)	2,303,049 (2.15)
	DIY^a^ mention	"* DIY *", "DIY *", "* DIY,*", "DIY,*"	1288 (4.59)	1,183,187 (1.11)
				
**Product types**				
	Starter kit	"*starter kit*", "*starterkit*"	3023 (10.76)	9,805,395 (9.17)
	Disposable	"*disposable*"	1263 (4.50)	4,710,782 (4.40)
	E-hookah	"*hooka*", "*shisha*", "*eshish*"	667 (2.37)	4,618,779 (4.32)
	E-cigar	"*cigar *"	746 (2.66)	1,203,781 (1.13)
	Mods (modifications)	"* mod *", "* mods *", "mod *", "* mod,*", "mod,*", "mods *", "* mods,*", "mods,*"	3946 (14.05)	9,999,591 (9.35)
	Cartomizer	"*cartomizer*"	2157 (7.68)	7,681,730 (7.18)
	Atomizer	"*atomizer*"	3631 (12.93)	10,161,207 (9.50)
	Cartridge	"*cartridge*"	2774 (9.88)	11,790,246 (11.02)
	Battery	"*battery*"	3338 (11.88)	15,013,078 (14.04)
	E-liquid	"*juice*", "*liquid*"	7637 (27.19)	25,626,650 (23.96)
	Refill	"*refill*"	2650 (9.43)	8,355,835 (7.81)
	Flavor	"*flavor*"	2979 (10.61)	7,754,066 (7.25)
	Nicotine free	"*zero nicotine*", "*nicotine free*", "*no nicotine*", "*without nicotine*", "*nicotinefree*"	192 (0.68)	1,273,715 (1.19)
Dual use				
	Marijuana	"*weed*", "*marijuana*"	909 (3.24)	2,993,990 (2.80)
**Brands (sales rank)**				
	Blu (1)	"* blu *", "*blucig*", "blu *", "* blu,*", "blu,*"	3507 (12.49)	11,764,026 (11.00)
	NJOY (2)	"* njoy*", "njoy*"	1235 (4.40)	7,460,512 (6.97)
	Mistic (3)	"*mistic*"	55 (0.20)	159,961 (0.15)
	21st Century Smoke (4)	"*21st cent*", "*21stcentury*", "*21 cent*", "*21century*"	223 (0.79)	124,381 (0.12)
	Logic (5)	"* logic *", "logic *", "* logic,*", "logic,*"	263 (0.94)	326,800 (0.31)
	Finiti (6)	"* finiti *", "finiti *", "* finiti,*", "finiti,*"	142 (0.51)	47,246 (0.04)
	Nicotek (7)	"*nicotek*"	40 (0.14)	14,551 (0.01)
	Cigirex (8)	"*cigirex*"	1 (0)	453 (0)
	Cig20 (9)	"*cig20*"	2 (0.01)	15 (0)
	Green Smart Living (10)	"*green smart*", "*greensmart*"	16 (0.06)	17,833 (0.02)
**Top 10 best-selling brands**			4280 (15.24)	15,678,220 (14.66)
	eGo	"* ego *", "ego *", "* ego,*", "ego,*"	3103 (11.05)	10,191,677 (9.53)
	V2	"* v2 *", "v2 *", "* v2,*", "v2,*"	2783 (9.91)	5,386,900 (5.04)
	Vuse	"*vuse*"	6 (0.02)	12,002 (0.01)
	MarkTen	"*markten*"	2 (0.01)	58 (0)
	Green Smoke	"*green smoke*"	1281 (4.56)	4,813,150 (4.50)
	Joyetech	"* joye *", "*joyetech*", "joye *", "* joye,*", "*joye,*"	2395 (8.53)	12,742,376 (11.91)
	Volcano	"*volcano*"	1891 (6.73)	4,887,673 (4.57)
	LavaTube	"*lavaTube*", "*lava tube*"	1205 (4.29)	3,276,914 (3.06)
Any brands			9379 (33.39)	38,240,598 (35.75)

^a^DIY: do it yourself.

**Table 4:** YouTube account activity for top-selling e-cigarette brands as of June 30, 2013.

Brand	Account name	Videos posted, n	View count, n	Subscribers, n	Collected videos, n
Blu	BluCigs	32	463,157	1510	32
NJOY	NJOYeCigs	3	279,736	279	8
Mistic	MisticEcigs	8	868	6	2
21st Century Smoke	21stCenturySmokeECig	1	1196	0	1
Logic	LogicDisposableEcigs	8	2262	28	8
Finiti	MyFiniti	13	22,871	24	6
Nicotek	Nicotekecigs	30	7284	15	31
Cigirex	CigirexUK	1	580	0	0
Green Smart Living	DeanGreenSmart	2	598	3	1

## Discussion

### Principal Findings

The regulatory status of e-cigarettes has major implications for their marketing and promotion. In 2008, a federal district court ruled that the FDA could not regulate e-cigarettes as drugs or devices unless they were marketed for therapeutic purposes (ie, smoking cessation). This ruling was later upheld by the US Court of Appeals. However, the appeals court clarified that e-cigarettes may be subject to regulation as “tobacco products” since they contain nicotine, which is derived from tobacco [21]. Such regulation could make e-cigarettes subject to the marketing guidelines that govern traditional tobacco products, including ingredient listing; good manufacturing practice; mandated health warnings; and prohibitions against television and radio advertising, event sponsorship, and youth-targeted advertising [39]. In April 2014, the FDA issued a proposed rule to deem electronic cigarettes, among other products, as tobacco products. If finalized, the FDA’s proposed Deeming Rule would extend FDA regulatory authority to e-cigarettes and other tobacco products, allowing the FDA to propose rules that restrict the manufacture, distribution, and marketing of e-cigarettes, including advertising and promotion restrictions. Until then, e-cigarette manufacturers and distributors can continue to make relatively unrestricted advertising appeals. Despite prohibitions, promotional claims about the role of e-cigarettes in smoking cessation may be common and may have contributed to increased e-cigarette use [23]. On YouTube, over 2500 e-cigarette videos mention smoking cessation in text fields and thus may be retrieved by consumer searches related to quitting.

Our analyses suggest that YouTube is heavily utilized for promotional and networking purposes, with 70% of videos including Web addresses; however, the platform appears to be very unevenly utilized for brand-specific promotion. For example, we noted that only 15% of videos included mentions of the top 10 best-selling brands, with only Blu and NJOY representing greater than 1% of total videos or views. Further, the vast majority of brand mentions were not made through company accounts. For example, videos from the account BluCigs accounted for only 32 videos and approximately 500,000 views, representing 0.8% of total videos mentioning Blu and 4% of total views. Videos uploaded by NJOYecigs accounted for 0.3% of videos mentioning NJOY and for 4% of total views. Efforts by some vendors, nonetheless, had potential to change the landscape of available content; one website, EcigsFreeTrialOffer.com (no longer active), accounted for 80% of new videos posted in April 2012. This spike coincided with the launch of the first *Tips from Former Smokers* campaign, a major antismoking media effort sponsored by the US Centers for Disease Control and Prevention. Keyword searches for additional brands revealed variable presence on YouTube: following Blu, eGo and V2 comprised the second and third highest fraction of content. Most e-cigarette liquids contain 6, 12, 18, or 24 mg/mL nicotine levels, but concentrations of 36 mg/mL and 100 mg/mL solutions for making e-liquid also are available [40]. Given this wide variability in nicotine delivery and manufacturing standards for e-cigarettes, heavy promotion of certain brands may lead to use of devices not optimized for nicotine delivery and thus ineffective for smoking cessation. It is unknown how marketing appeals of smaller brands may differ from those of larger companies.

Our results show a high level of user engagement with e-cigarette content, with over 100 million total views for e-cigarette-related videos as of June 2013. To put this finding into context, the e-cigarette TV ads reached 29 million youth and young adults in 2013 [41]. Furthermore, 43% of videos included the keyword *review* and 10% included keywords indicating product demonstrations, both of which suggest videos originating from consumers or affiliated marketers. Mentions of mods, atomizers, e-liquids, and marijuana suggest that customization plays a large role in e-cigarette discourse on YouTube. Consumers’ ability to choose and manipulate aspects of their e-cigarette experience, including flavor, nicotine content, and battery, may have contributed to their rising popularity, but also raises questions about uneven efficacy in tobacco replacement and the potential gateway to other substance abuse. The availability of flavored juices has been criticized by some, since they may appeal to nonsmokers, including younger consumers [42].

In a sample of 280 e-cigarette videos, we found none to be age restricted by YouTube, indicating that youth can easily view and access e-cigarette videos on YouTube. Since the vast majority of these videos provide links to vendors or branded websites, these videos may enhance opportunities for underage e-cigarette purchase.

While e-cigarette marketing efforts largely leverage new media channels, traditional media plays an increasing and interconnected role. Cigarette advertising has been prohibited from US television and radio since 1971, but in recent years, e-cigarette brands have introduced television-advertising campaigns [43]. Television advertisements may use many strategies employed by cigarette advertisers in the past, including jingles, celebrity endorsers, and mascots [44]. Television advertising may also drive activity on social media channels, including YouTube. For example, we noted that a UK advertisement for E-lites garnered an additional 2 million views when posted on YouTube. Several studies have noted additional television content, including footage of actress Katherine Heigl smoking an e-cigarette on The Late Show with David Letterman and a clip from the program The Doctors [37]. Our search for a list of several celebrities featured in viral and traditional marketing campaigns (ie, Katherine Heigl, Stephen Dorff, Bruno Mars, and Courtney Love) retrieved only 153 unique videos, yet these videos were associated with view counts almost four times higher than average, comprising over 2 million total views.

### Limitations

Our study has several limitations and raises questions for further research. First, the study relies on keywords to capture and categorize content relevant to e-cigarettes, and any set of keywords is necessarily incomplete since new brands and terminology are continually emerging. In particular, we may have overlooked some non-English e-cigarette keywords as well as variations of the slang term *vape*. As a result, our estimates of e-cigarette videos and their view counts underestimate their true overall presence and impact on YouTube. However, given that the vast majority of videos in our dataset include multiple e-cigarette keywords, we believe we likely captured the majority of relevant content. We cannot exclude the possibility that there are YouTube videos that discuss e-cigarettes but do not reference them in any metadata field, but such videos would be unlikely to represent influential content, since attracting viewers relies on effective retrieval of videos by e-cigarette-related search queries.

We also used keyword rules to characterize themes within e-cigarette videos; again, this method is likely to underestimate the true presence of these themes. Further, we did not analyze variations of messages within each theme, for example, to characterize differences between various health claims or to investigate mentions of smoking-related disease. The keyword query rules applied were simple and thus not sensitive to context, in either the video content or the metadata. We did not undertake content analysis of the videos themselves, a task that fell outside the scope of this study but may have yielded rich results. Certain content elements are of considerable interest, but were not discernible by our methodology; for example, we could not reliably distinguish between promotional efforts and noncommercial consumer perspectives. Even with review of video content, such categorization would be challenging given that those affiliated with e-cigarette brands or companies may represent themselves as consumers as a marketing strategy [5,19].

More work is required to discern whether health, safety, and commercial claims derive from commercial or individual accounts. Characterizing commercial claims may clarify social media marketing guidelines. Identifying consumer experiences may help clarify whether and how e-cigarettes are used for smoking cessation, and thus contribute to public health efforts to optimize these products’ harm reduction potential. Characteristics of influential users may be explored by examining posts and comments data for individual accounts [45]. Finally, we did not examine targeted advertising that accompanies searching for and viewing content on YouTube, which may be another important way in which consumers are exposed to e-cigarette-related content [9].

### Conclusions

In summary, our study provides an approximation of the total amount of content and consumer engagement on YouTube related to e-cigarette use. Our analyses suggest uneven use of YouTube for promotional purposes by e-cigarette brands, and a high level of engagement with a small subset of content. Further research is needed to establish the information contained in e-cigarette-relevant YouTube videos and how these videos impact consumers’ attitudes, beliefs, and risk perceptions about e-cigarettes and subsequent decisions regarding use of e-cigarettes, conventional cigarettes, and evidence-based smoking cessation aids. A better understanding about the extent, content, and impact of e-cigarette YouTube videos can aid the public health community and policymakers to ensure appropriate e-cigarette marketing regulations on social media platforms.

## Appendix

Multimedia Appendix 1 E-cigarette keyword rules.

Multimedia Appendix 2 Metadata search terms.

## Abbreviations

CTP: Center for Tobacco Products
DIY: do it yourself
ENDS: electronic nicotine delivery systems
FDA: Food and Drug Administration
NCI: National Cancer Institute
NIH: National Institutes of Health
