# Supplementary material for: 100 Million Views of Electronic Cigarette YouTube Videos and Counting: Quantification, Content Evaluation, and Engagement Levels of Videos
Source: J Med Internet Res. 2016 Mar 18;18(3):e67. doi: 10.2196/jmir.4265 (PMC4818373; doi:10.2196/jmir.4265)
Supplement: Multimedia Appendix 1 [file jmir_v18i3e67_app1.pdf]

e cig
e cig review
e cig smoking
e cig tricks
e cig commercial
e cig how it works
e cig refill
e cig video
e cig starter kits
e cig company
e cig mod
e cigar
e cigar review
e cigar smoking
e cigar tricks
e cigar commercial
e cigar how it works
e cigar refill
e cigar video
e cigar starter kits
e cigar company
e cigar mod
e cigarette
e cigarette review
e cigarette smoking
e cigarette tricks
e cigarette commercial
e cigarette how it works
e cigarette refill
e cigarette video
e cigarette starter kits
e cigarette company
e cigarette mod
electronic cigar
electronic cigar review
electronic cigar smoking
electronic cigar tricks
electronic cigar commercial
electronic cigar how it works
electronic cigar refill
electronic cigar video

electronic cigar starter kits
electronic cigar company
electronic cigar mod
electronic cigarette
electronic cigarette review
electronic cigarette smoking
electronic cigarette tricks
electronic cigarette commercial
electronic cigarette how it works
electronic cigarette refill
electronic cigarette video
electronic cigarette starter kits
electronic cigarette company
electronic cigarette mod
e cig NJOY
e cig Blu
e cig 21st Century
e cig Krave
e cig Logic
e cig V2
e cig Green Smoke
e cig The Safe Cig
e cig Pure Cigs
e cig White Cloud

e cig Smoketip
e cig South Beach Smoke
e cig Eversmoke
e cig Eluma
e cig Zerocig
