# Supplementary material for: 100 Million Views of Electronic Cigarette YouTube Videos and Counting: Quantification, Content Evaluation, and Engagement Levels of Videos
Source: J Med Internet Res. 2016 Mar 18;18(3):e67. doi: 10.2196/jmir.4265 (PMC4818373; doi:10.2196/jmir.4265)
Supplement: Multimedia Appendix 2 [file jmir_v18i3e67_app2.pdf]

"*atomizer*"; "*cartomizer*"; "*blu cig*"; "*njoy cig*"; "*green smoke*"; "*south beach smoke*"; "*ehookah*"; "*e-hookah*"; "*ejuice*"; "*e-juice*"; "*eliquid*"; "*e-liquid*"; "*esmoke*"; "*e-smoke*"; "*eversmoke*"; "*joye 510*"; "*joye510*"; "*joyetech*"; "*lavatube*"; "*smartsmoker*"; "*smokestik*"; "*v2 cig*"; "*v2cig*"; "zerocig*"; "*zero cig*"; "*vape*"; "*vapin*"; "* e cig*"; "*ecig*"; "*e-cig*"; "*electronic cig*"; "*smoke free cig*"; "*ego-c*"; "*ego-t*"; "smokefree cig*";

"*e-zig*"; "*eshish*"; "* njoy *"; "*smokeless cig*"; "*logic cig*"; "*electroniccig*"; "*electric cig*"; "* e hookah*"; "*smokepass*"; "*vapor cig*"; "*e-lites*"; "*e- cig*"; "*sigarette ele*"; "* e.cig*";

"* v2 *"; "*vapor nine*"; "*vapestick*"; "*mountain vapor*"; "*clearomizer*"; "*vaporchase*"; "*provape*"; "*vapour cig*"; "*ciga-tech*"; "*ovale*"; "*green cig*"; "*greencig*"; "* vapor *";

"* joye*"; "* finiti *"; "* nicotek *"; "* cigirex *"; "* cig20 *"; "*green smart*"; "*greensmart*";

"* vuse *"; "* markten *"; "* BluCigs *"
